# Physical Activity across Retirement Transition by Occupation and Mode of Commute

**DOI:** 10.1249/MSS.0000000000002326

**Published:** 2020-03-09

**Authors:** ANNA PULAKKA, TUIJA LESKINEN, KRISTIN SUORSA, JAANA PENTTI, JAANA I. HALONEN, JUSSI VAHTERA, SARI STENHOLM

**Affiliations:** 1 Department of Public Health, University of Turku and Turku University Hospital, Turku, FINLAND; 2 Centre for Population Health Research, University of Turku and Turku University Hospital, Turku, FINLAND; 3 Finnish Institute for Health and Welfare, Helsinki, FINLAND; 4 Department of Public Health, Faculty of Medicine, University of Helsinki, Helsinki, FINLAND; 5 Stress Research Institute, Stockholm University, Stockholm, SWEDEN

**Keywords:** ACCELEROMETER, RETIREMENT TRANSITION, OCCUPATION, TRANSPORT, COMMUTING

## Abstract

**Purpose:**

Retirement induces changes in the composition of daily physical activity. Our aim was to examine changes in accelerometer-measured physical activity around transition to statutory retirement among men and women by occupational category and by preretirement modes of commuting.

**Methods:**

We included 562 workers (mean [SD] age, 63.3 [1.1] yr; 85% women) from the Finnish Retirement and Aging study. The participants wore an accelerometer on their nondominant wrist for 1 wk before and 1 wk after retirement, with 1 yr between the measurements. We compared mean daily activity counts before and after retirement between manual and nonmanual occupations by gender and by preretirement commuting mode using linear models with generalized estimating equations.

**Results:**

Before retirement, women were more active than men (2550 (95% confidence interval, 2500–2590) vs 2060 (1970–2140) mean daily activity counts), with the most active group being women in manual occupations. After retirement, physical activity decreased by 3.9% among women and increased, albeit nonsignificantly, by 3.1% in men. The decrease was most pronounced among women in manual and increase among men in nonmanual occupations. After retirement, women remained more active than men (2450 (95% confidence interval 2390–2500) vs 2120 (2010–2230) counts). Active commuting, especially cycling, before retirement was associated with higher physical activity both before and after retirement, and these people also maintained their total activity lever better than did those who commuted by public transportation.

**Conclusions:**

Although women in manual occupations decreased and men in nonmanual occupations increased their activity after retirement, women were more active than men both before and after retirement. Those who engaged in active commuting before retirement maintained their activity level also after retirement.

## Abstract 2

Supplemental digital content is available in the text.

Retirement is one of the major life transitions, resulting in changes in time availability and daily routines, which may induce changes in physical activity (1,2). Physical activity takes place in different domains of which the most commonly referred to are occupational, leisure time, domestic, and transportation (3), where the last one can further be specified into commuting or other transportation. After retirement, time used for work and commuting is replaced by behaviors that can either increase or decrease total physical activity.

Few previous studies have examined changes in leisure-time physical activity by following people over retirement transition with varying results. An increase in leisure-time physical activity after retirement was found in some (2–10), but not in all (1,11,12) studies. However, all the previous longitudinal studies have measured physical activity by self-reports, a crude measure subjective to reporting bias. They have also used a varying follow-up time, up to >10 yr (9,12), which makes separating retirement-related changes from aging-related changes challenging. In addition, most of the studies assessed leisure-time physical activity only (4–6,8–10), or a combination of leisure-time physical activity and transport-related physical activity (7,12), neglecting occupational physical activity and thus being unable to assess changes in total physical activity. The studies that have included occupational physical activity have reported either general increase (2) or decrease (3) in total physical activity or increase in total physical activity among those retiring from sedentary jobs and a decrease among those retiring from physically demanding jobs (11).

To the best of our knowledge, there are no previous studies examining longitudinal changes in accelerometer-measured total physical activity across retirement transition. Therefore, the aim of this study was to examine how accelerometer-measured total physical activity changes after transition to statutory retirement among men and women in manual and nonmanual occupations. In addition, we assessed the preretirement physical activity accumulated at work, during commuting, and during leisure time and examined changes in physical activity by preretirement modes of commuting.

## METHODS

### Setting and participants

The Finnish Retirement and Aging (FIREA) study is an ongoing longitudinal cohort study of older adults in Finland established in 2013 (7,13). The FIREA survey cohort included all public sector employees whose individual estimated retirement date was between 2014 and 2019, who were working in 1 of the 27 municipalities in Southwest Finland or in the 9 selected cities or 5 hospital districts around Finland in 2012, and who responded to at least one of the FIREA questionnaires by the end of 2018. Information on individual estimated retirement date was obtained from the pension insurance institute for the municipal sector in Finland (Keva). Participants were first contacted 18 months before their estimated retirement date by sending a questionnaire.

The Finnish-speaking FIREA survey participants whose estimated statutory retirement date was in 2016–2019, who had responded to the first questionnaire and who were still working, were eligible for the activity substudy (*n* = 2663). These participants were sent an invitation letter to participate in the activity substudy, and of them, 908 participants (34% of the eligible) returned the informed consent. There were slightly more women and employees with nonmanual occupation, and fewer self-reported inactive people, among the participants who consented to the accelerometer measurement as compared with those who were eligible, but did not consent (13). We followed the participants up annually with questionnaires and accelerometer measurements and enquired about the timing of retirement in each phase of the data collection. Between September 2014 and April 2019, 575 participants had used the accelerometer at least once before and once after retirement, with 1 yr in between the measurements. The rest of the participants were not yet retired (*n* = 321) or did not use the accelerometer (*n* = 12) and were therefore not included in the current analyses. The FIREA study is conducted in line with the Declaration of Helsinki and was approved by the Ethics Committee of Hospital District of Southwest Finland. The participants provided written informed consent before participation.

### Activity measurements

Physical activity was measured over 7 consecutive days and nights with triaxial ActiGraph wActiSleep-BT and wGT3X-BT accelerometers (ActiGraph, Pensacola, FL). Participants were instructed to wear the device on their nondominant wrist at all times, including during water-based activities such as swimming, but to remove it for sauna. Data were collected during all seasons (25% spring, 10% summer, 35% fall, 30% winter). Data from the accelerometers were downloaded and converted into 60-s epochs in ActiLife software, version 6.13 (ActiGraph). We used vector magnitude (VM) counts per minute (CPM), which were calculated as the square root of the sum of squared activity counts of the three axes.

We included wear time between the first and last recorded times in the participant log and excluded nonwear time using the algorithm developed by Choi et al. (14) and sleep time by the algorithm available in the ActiLife software (15,16). A valid day of measurement was defined as minimum of 10 h of wake wear time. According to a commonly used criteria (17), we excluded those participants who had less than four valid measurement days either before or after retirement (*n* = 13), leaving 562 participants for the analyses. From analyses regarding mode of commuting, we also excluded 89 participants who had no information on commuting mode, leaving 473 participants in these analyses. When assessing preretirement activity accumulated during work, commuting, and leisure time, we further excluded participants who did not have information of working and commuting time, thus including 434 participants in these analyses.

In a log accompanying the accelerometers, the participants were asked to report information about preretirement working day (working day or day off) and, for working days, start and end times of each work shift as well as duration and mode of commuting. We combined the data from the logs with the accelerometer data and identified work time by using reported start and end times of each work shift. Commuting was defined by splitting reported commuting time into two and assigning one-half for time period before start of the work shift and the other half for time period after end of the work shift. The rest of the wake wear time (leisure time during working days and days off) was defined as leisure time, which in the case of our accelerometer data might include a mix of recreational, transport, domestic, and incidental physical activity as well as sedentary time.

We used three measures of physical activity for wake wear time activity. First, we used mean daily activity counts (mean of daily mean VM CPM [18]) to compare overall physical activity before and after retirement, and the change after retirement, by gender and occupational groups. Second, we used mean hourly activity counts (mean of hourly mean VM CPM of each hour of the day [13]) to visualize activity levels across the day before and after retirement by gender. Third, proportion of total daily activity counts (mean of daily sum of VM CPM [19]) accumulated at work, and during leisure and commuting time, and postretirement total daily activity counts were determined to examine activity accumulated at different preretirement domains between gender and occupational groups. Weekly mean of total daily activity counts for each time slot (work, commuting, and leisure time) was calculated as (5 × working day + 2 × day off)/7. As a *post hoc* analysis to further explore activity during commuting, we also calculated the mean activity counts during preretirement commuting by mode of commuting.

### Assessment of gender, occupational category, and commuting mode

We obtained participants’ gender, date of birth, and occupational title codes from the pension insurance institute. Occupational titles were categorized by the International Standard Classification of Occupations (ISCO) into manual (ISCO classes 5–9, e.g., practical nurses, cooks, maintenance workers, and cleaners) and nonmanual (ISCO classes 1–4, e.g., physicians, teachers, registered nurses, and secretaries) occupational categories (20).

Preretirement commuting mode was defined based on the information reported in the participant logs. First, we classified the mode of commuting for each day as a car (those who reported using car, and possibly also walking or cycling), public transport (those who reported a public transport method, e.g., a bus or a train, but possibly also other methods, such as walking or a car), walking (those who reported only walking), cycling (those who reported only cycling), or other (not falling into any of the aforementioned categories). Second, we classified the participants into five groups based on their most common mode of commuting (more than 50% of reported commuting days): car, public transport, walking, cycling, or mixed (no commuting mode exceeding 50% of the reported days). The group of mixed was excluded from the analyses because of the small number (*n* = 12).

### Assessment of covariates

Prevalent doctor-diagnosed cardiovascular diseases (angina pectoris, myocardial infarction, or cerebrovascular disease), musculoskeletal diseases (osteoarthritis, osteoporosis, sciatica, fibromyalgia, and rheumatoid arthritis), and diabetes, as well as self-reported mobility limitation (difficulty in climbing one flight of stairs or walking several blocks) (21), were derived from the preretirement questionnaire. We calculated body mass index (BMI) from self-reported weight and height (in kilograms per meter squared).

### Statistical analysis

We compared mean daily activity counts between men and women in different occupational categories and between preretirement commuting mode categories using ANOVA. We also used ANOVA to compare mean activity counts during commuting time and proportion of total daily activity counts accumulated from commuting between modes of commuting. The changes in activity across retirement were assessed using linear models with generalized estimating equations. The generalized estimating equation model takes into account the intraindividual correlation between the measurements. We constructed two models: model 1 adjusting for occupational category (for gender and commuting mode analyses), age, and duration of wake wear time of the accelerometer, and model 2 adjusting additionally for chronic diseases (yes/no), mobility limitation (yes/no), and BMI (continuous). We also tested the interaction effects between occupational category and time on mean daily activity counts separately among men and women. The statistical analyses were performed using SAS version 9.4 (SAS Institute, Inc., Cary, NC).

We created two types of graphs: First, to visualize activity patterns across the day, both before and after retirement, we present the mean hourly activity counts for each 24 h of the day by gender, excluding hours with less than 60 min of accelerometer measurement (<2% of the hours) but including sleep time. Second, to visualize the preretirement proportion of the total daily activity counts accumulated during work, commuting, and leisure time, and total postretirement physical activity, we present total daily activity counts from these different domains as bar charts by gender and occupational category.

## RESULTS

Table 1 presents characteristics of the participants by gender before retirement. Mean age of the 562 participants was 63.3 yr (SD 1.1), and a majority of them were women (85%). Two-thirds of the participants worked in nonmanual occupations, half of them had at least one chronic disease, whereas mobility limitations were rare. Car was the most common commuting mode for both men and women. Women commuted by car less than did men, but used public transport, walked, or cycled more than did men. Men had longer daily wake wear time for the accelerometer than did women.

**TABLE 1:**
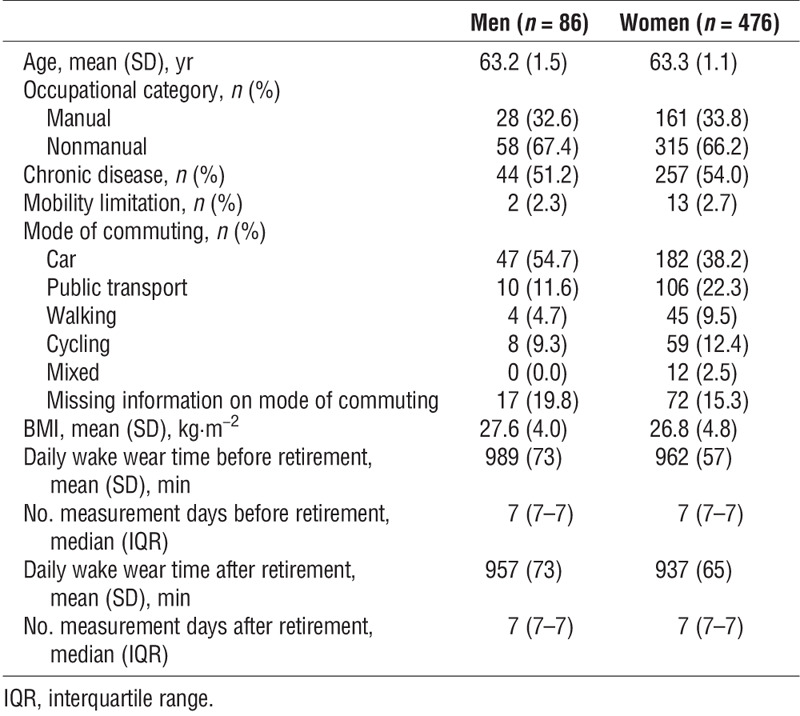
Characteristics of the participants before retirement.

Before retirement, the mean daily activity counts were higher in women than in men and in manual than in nonmanual occupations when adjusted for age and wake wear time (Table 2). In general, women decreased their daily activity after retirement (*P* value for time interaction = 0.001), whereas there was no change in men’s activity (*P* value for time interaction = 0.63; Table 2). Specifically, women in manual occupations decreased their activity by 9.5% (95% confidence interval (CI), −12.2% to −6.8%); however, no change was observed for women in nonmanual occupations (−0.3%; 95% CI, −2.2% to 1.6%). In contrast, there was no change in activity for men in manual occupations (1.0%; 95% CI, −5.9% to 7.8%) after retirement, but men in nonmanual occupations increased their physical activity by 5.0% (95% CI, 0.4%–9.7%). After retirement, women in both occupational categories were more active than men in manual or nonmanual occupations. All the results remained similar in the models adjusted additionally for preretirement chronic diseases, mobility limitation, and BMI.

**TABLE 2:**
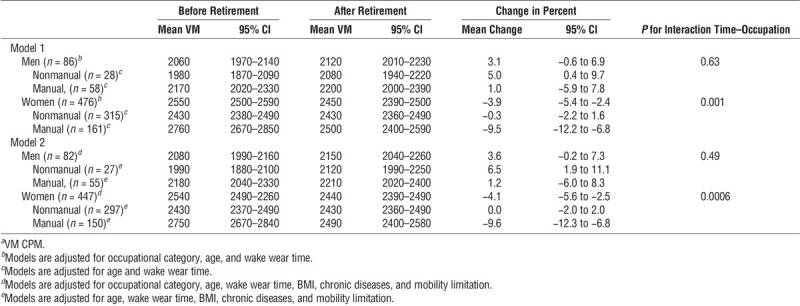
Mean daily activity counts*^a^* and their 95% CI before and after retirement and the change in mean daily activity counts.

**FIGURE 1:**
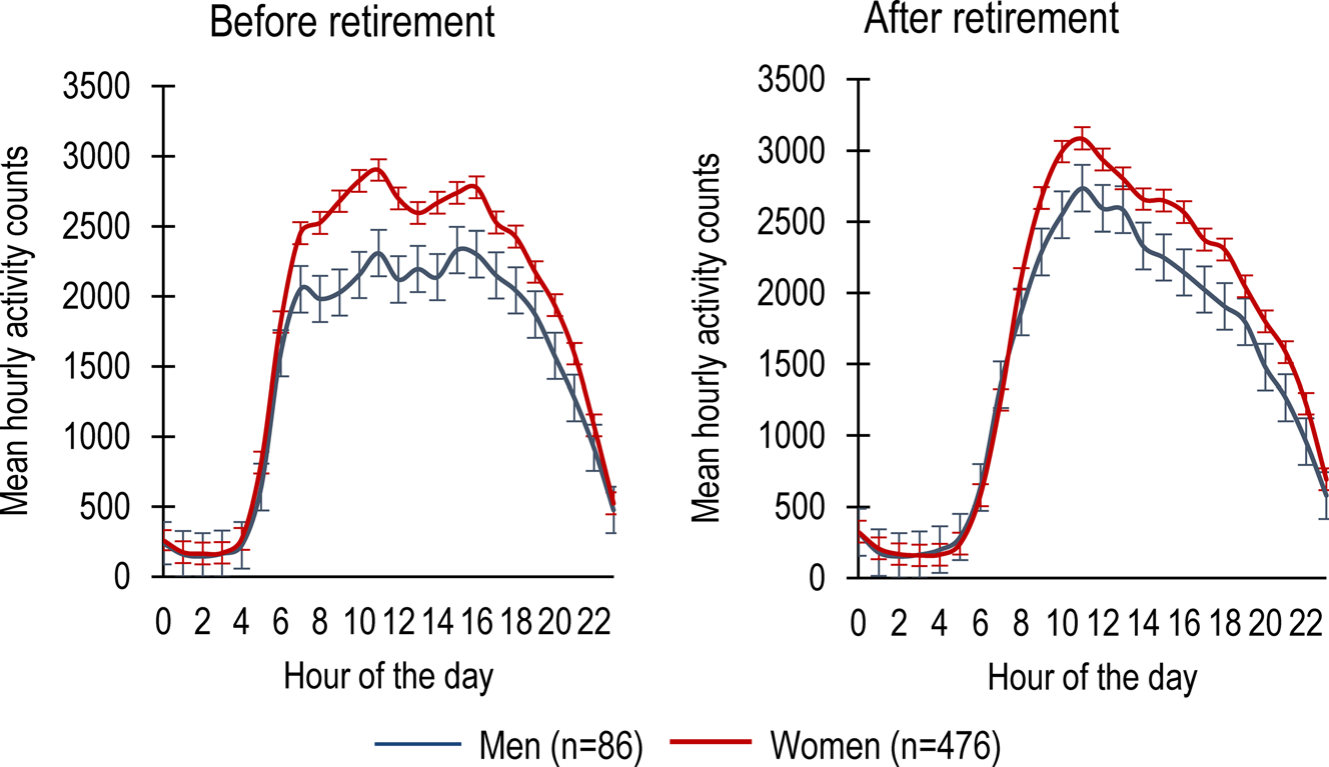
Twenty-four-hour activity patterns before and after retirement for men and women.

Figure 1 depicts the shape of the daily activity patterns before and after retirement for both women and men. Before retirement, activity was initiated earlier in the morning and there were two activity peaks in the day, whereas after retirement, activity was initiated later and had one peak during midday. Decline in activity in the evenings was relatively similar before and after retirement.

**FIGURE 2:**
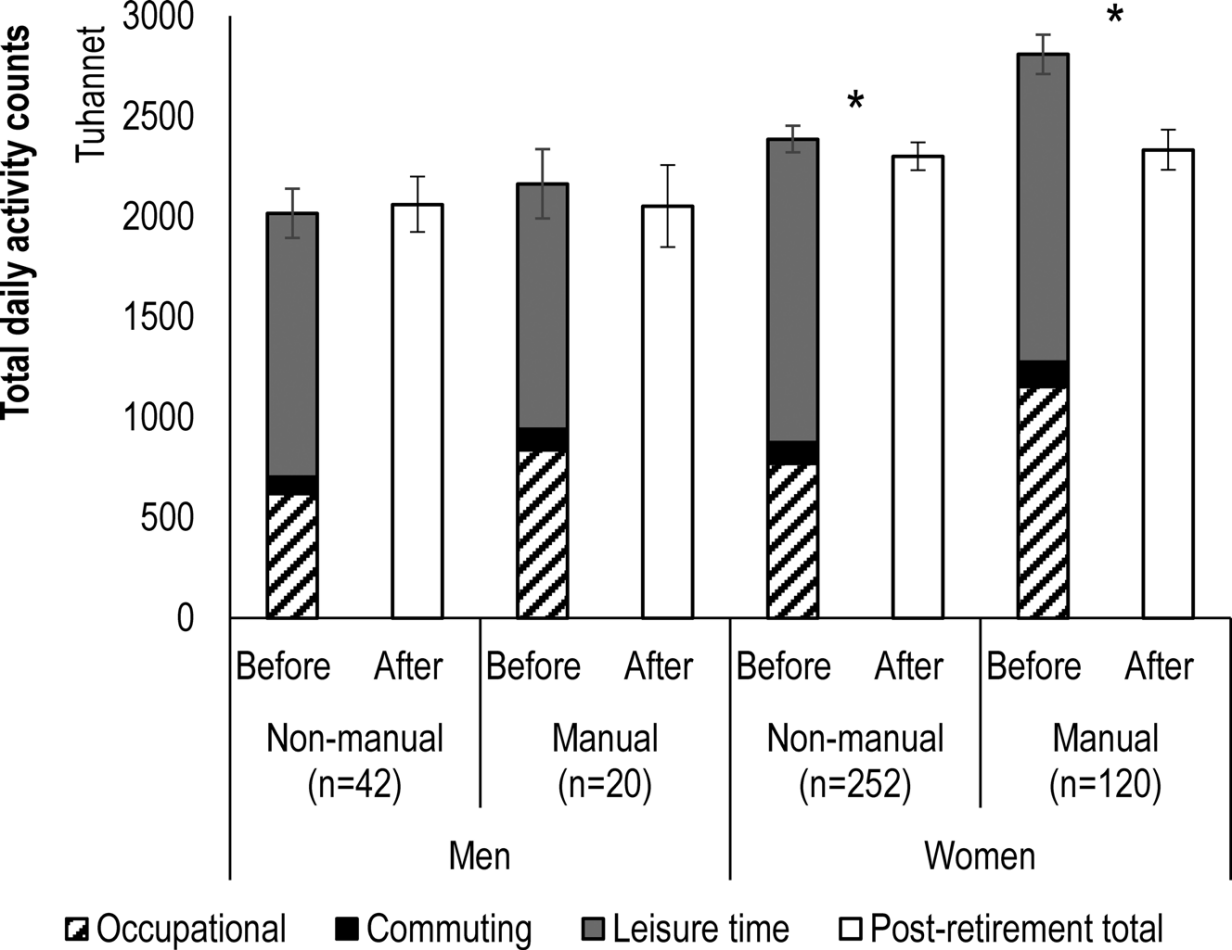
Total activity counts before and after retirement. *Statistically significant difference between measurements.

Distribution of preretirement daily activity to different activity domains was relatively similar for men and women (Fig. 2). When examined by occupational category, work constituted a larger, and leisure time a smaller, proportion of total daily activity in manual workers compared with nonmanual workers. No marked difference between the occupational groups was observed in the proportion of commuting.

Finally, we examined changes in physical activity by preretirement commuting mode. In general, those who walked or cycled to work were more active than car drivers before retirement and they also maintained their activity level after retirement (Table 3). The only group that markedly changed their mean daily activity counts after retirement was public transport users, who decreased their activity by 6.1% (95% CI, −9.4% to −2.9%). After retirement, cyclists were still more active than those who had driven to work, and more active than those who had used public transport for commuting. The results remained robust in the fully adjusted models. When examining commuting time specifically, those walking to work had the highest mean activity counts during commuting adjusting for gender, occupation and age (4910; 95% CI, 4600–5230), followed by cyclers (3540; 95% CI, 3270–3810; see Table, Supplemental Digital Content 1, mean activity counts during commuting and accumulation of total daily activity counts from commuting domain when adjusted for gender, occupation, and age, http://links.lww.com/MSS/B947). Those who used a car (3070; 95% CI, 2920–3220) or public transport (2990; 95% CI, 2780–3190) had the lowest activity counts during commuting. Participants commuting by public transport accumulated a higher proportion of their physical activity from commuting domain than car drivers and cyclers, whereas those walking to work accumulated a higher proportion from commuting than the car drivers.

**TABLE 3:**
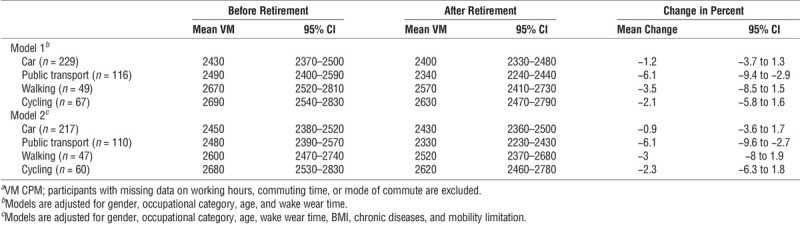
Mean daily activity counts*^a^* and their 95% CI before and after retirement and the change in mean daily activity counts by preretirement mode of commuting.

## DISCUSSION

In this longitudinal study of within-individual changes in accelerometer-measured physical activity across retirement transition, we observed that women who retired from manual occupations decreased, and men in nonmanual occupations increased their physical activity after retirement. The daily activity patterns showed two activity peaks during daytime before retirement but only one activity peak after retirement. We also observed that active commuting before retirement was associated with higher physical activity both before and after retirement, and people engaged in active commuting also maintained their total activity level better than did those who commuted by public transportation.

To the best of our knowledge, this is the first study to measure within-individual changes in accelerometer-measured physical activity across retirement transition. Although in general there was no major change in mean daily activity counts, we observed that certain groups changed their activity after retirement. Women in manual occupations and employees who had used public transport for commuting decreased their physical activity. This is in accordance with an American study where self-reported total physical activity decreased for those who retired from physically demanding jobs (11). As occupational physical activity accounts for a marked proportion of total physical activity (22) and commuting by public transport includes physical activity (23,24), it is plausible that exiting the workforce reduces total physical activity for those who have accrued high amount of their physical activity during work or commuting. However, in other circumstances, occupational physical activity may be harmful for health (25,26), thus decreasing it or replacing it with leisure-time physical activity after retirement may be beneficial.

Although there was no significant change in physical activity among men in total, those in nonmanual occupations increased their physical activity after retirement. Possible reasons for increasing physical activity after retirement include replacing working day routines with new routines that include physical activity, desire to seek new challenges after finishing work, and expected health benefits of physical activity (27). There was no increase in physical activity among women. This agrees with a Japanese study where an increase in leisure-time physical activity during retirement transition was higher among men than women (10). However, it is possible that the women in our study also increased their leisure-time physical activity, although the increase was not enough to cover the decrease resulting from removal of occupational and commuting physical activity.

In our study, women, especially those in manual occupations, were more active than men before and after retirement. The least active, both before and after retirement, were men in nonmanual occupations. These gender differences may arise from differences in occupations (28), more common active commuting among women than men (29), or household chores, which are more commonly taken up by women especially in this age group (30).

Commuting accounted for about 4%–6% of total daily activity counts in our study, with the highest contribution of commuting for those who walked and used public transport. This is in line with some previous studies showing that both walking and using public transport for commuting contribute more to total physical activity than commuting by car, although the contribution was less in our study compared with the previous studies (24,31). Although the contribution was modest, those who used active transport for commuting were more active than those who used car or public transport before retirement. Interestingly, cyclers remained more active than car commuters also after transition to retirement. There are some possible explanations for this. First, cycling might be a proxy of an active lifestyle and those who cycle to work continue to be active in their daily lives after retirement. In a qualitative review of Barnett et al. (27), retirees considered lifelong participation in physical activity as a prerequisite for maintaining physical activity while retired. Second, it is possible that cyclers live in an area where they can use active transport for other errands than commuting. Based on a previous Finnish study, people living in areas with close distance to business districts are more likely to commute by walking or cycling (32). Our results support earlier findings that active transport before retirement is associated with higher total physical activity (24,31) and might be a way to increase physical activity also after exiting the workforce.

The main strengths of the study include accelerometer assessment of total physical activity before and after retirement with 1-yr interval enabling detailed examination of within-individual changes across retirement transition. The relatively large study population consisted of participants representing a wide variation of occupations. In addition, we were able to distinguish between work, commuting and leisure time in the accelerometer measurements before retirement and study the changes in total physical activity by preretirement commute domains. The measurements of each participant were done at approximately same time of the year; thus, seasonal differences are unlikely to confound our results.

The study has some weaknesses that should be considered when interpreting the results, many of which are related to our measure of commuting time. Because accelerometers only detect movement of the part of the body where they are attached to, wrist-worn accelerometers may underestimate activity when cycling (33). This is reflected in the lower activity counts for cyclists than walkers during commuting time. However, our study participants who cycled to work were generally as active as those who walked to work, which might be due to the cyclists having an active lifestyle also other times of the day. In addition, we observed some seasonal differences in the commuting modes: there was less cycling (5% vs 18%) and more driving (54% vs 48%), use of public transport (29% vs 24%), and walking (12% vs 10%) during winter than during other seasons. It is thus possible that we have misclassified some people whose activity measurements were on winter and who would be active cyclists during other seasons. Furthermore, our measure of commuting time has some uncertainties. There is a possibility of misclassifying participants who had multiple commuting modes, although we included only participants who reported over 50% of days commuted by one mode. Furthermore, it is possible that some of the participants did not start their commute immediately at the end of their working day, or they may have run errands on their way to or from work. However, we observed that people walking for commute had the highest and people using car or public transport had the lowest activity counts during commuting time. This suggests that for the majority of cases we were able to identify the actual commuting time, although some uncertainty remains.

We used a relatively simple categorization by dividing daily activity before retirement into only three domains (work, commute, and leisure), because these domains were captured by the information from the daily logs. Another weakness of the study is that we did not have information on activity domains after retirement. Further studies, for example, using global positioning system that enables tracking activity locations, are needed to distinguish other domains, namely, transport other than commuting, domestic, and incidental physical activity. Furthermore, our study also had a short follow-up, only 1 yr, which facilitates the separation of retirement-related changes from aging-related changes but limits assessing if the changes in physical activity remain stable after retirement. Therefore, studies with longer follow-up are needed to assess long-term changes in physical activity after retirement. Moreover, because of some features of our setting, namely, employees of public sector with high proportion of women and infrastructure that allows walking and cycling to work, our results may not be generalizable to other countries and occupational sectors. In addition, it is important to highlight that the participants entered to statutory age-based retirement, and therefore are generally healthier compared with those who retire early, for example, based on disability or unemployment.

In conclusion, women in manual occupations decreased and men in nonmanual occupations increased physical activity after retirement. Despite this, women remained more active than men after retirement. Active commuting, especially cycling, before retirement was associated with higher physical activity both before and after retirement.

## Supplementary Material

SUPPLEMENTARY MATERIAL
