# Supplementary material for: Physical Activity across Retirement Transition by Occupation and Mode of Commute
Source: Med Sci Sports Exerc. 2020 Mar 9;52(9):1900–7. doi: 10.1249/MSS.0000000000002326 (PMC7431137; doi:10.1249/MSS.0000000000002326)
Supplement: SUPPLEMENTARY MATERIAL [file mss-52-1900-s001.pdf]

Physical activity across retirement transition by occupation and mode of commute

Pulakka A, Leskinen T, Suorsa K, Pentti J, Halonen JI, Vahtera J, Stenholm S

Table, Supplemental Digital Content 1. Mean activity counts during commuting and accumulation of total daily activity counts from commuting domain when adjusted for gender, occupation, and age.

Pre-retirement mode of commute	Mean VM	95% CI		% of total daily activity counts accumulated from commuting	95% CI	
Car (n=213)	3070	2920	3220	3.7	3.3	4.0
Public transport (n=112)	2990	2780	3190	5.7	5.2	6.2
Walking (n=46)	4910	4600	5230	5.0	4.2	5.8
Cycling (n=64)	3540	3270	3810	4.0	3.3	4.6

VM, vector magnitude; CI, confidence interval
